# Characterizing mitochondrial phenotypes and MERCS in aged human skeletal muscle myoblasts

**DOI:** 10.1371/journal.pone.0343604

**Published:** 2026-02-20

**Authors:** Yufu Unten, Kazuaki Takafuji, Yumiko Masukagami, Isshin Shiiba, Keigo Horiuchi, Filip Husnik, Shigeru Yanagi, Norifumi Tateishi, Toshihide Suzuki

**Affiliations:** 1 Research Institute, Suntory Global Innovation Center Limited, Kyoto, Japan; 2 Evolution, Cell Biology, and Symbiosis Unit, Okinawa Institute of Science and Technology, Okinawa, Japan; 3 Laboratory of Molecular Biochemistry, Department of Life Science, Faculty of Science, Gakushuin University, Tokyo, Japan; Fujita Health University: Fujita Ika Daigaku, JAPAN

## Abstract

Age-associated declines in skeletal muscle function are linked to cellular senescence and mitochondrial alterations, yet mitochondrial phenotypes in aged human myoblasts remain insufficiently characterized. Here, we examined primary skeletal muscle myoblasts from young and elderly donors to assess mitochondrial function, morphology, and mitochondria–endoplasmic reticulum (ER) contact sites (MERCS). Myoblasts from older donors exhibited senescence features, including elevated SA-β-gal activity and reduced Lamin B1 expression, accompanied by increased mitochondrial oxidative stress. Despite marked mitochondrial hyperfusion and increased mitochondrial DNA content, mitochondrial oxygen consumption rate and membrane potential per mitochondrial area were comparable between young and old cells. MERCS were significantly elevated in aged myoblasts and were reduced by scavenging mitochondrial reactive oxygen species (mtROS), indicating an association between oxidative stress and MERCS formation. These findings suggest that mitochondrial hyperfusion and enhanced MERCS accompany cellular aging in human myoblasts and may contribute to maintaining mitochondrial function under elevated oxidative stress.

## Introduction

As the proportion of elderly individuals in the global population continues to increase, there is an increasing urgency for the development of treatments and pharmaceuticals for age-related diseases. Among these, sarcopenia, a condition marked by an age-related decline in skeletal muscle mass, strength, and functionality, poses a significant threat to independent living in older adults [1]. Skeletal muscle is the most abundant tissue in the human body and plays crucial roles not only in autonomous movement, but also in metabolic and endocrine functions [2]. The age-related decline in skeletal muscle function is multifaceted and involves decreased physical activity, nutritional deficiencies, hormonal imbalances, and shifts in the balance between muscle protein synthesis and degradation [3,4]. Thus, the pathogenesis of sarcopenia remains unclear [3]. Skeletal muscle consists of muscle fibers formed by the proliferation, differentiation, and fusion of myoblasts [5]. Although mature skeletal muscles are relatively stable, excessive use or injury through exercise activates quiescent satellite cells, which differentiate into proliferative myoblasts. These proliferating myoblasts continue to undergo differentiation and fusion to repair damaged muscle fibers [6]. This muscle fiber repair mechanism declines with age, making it difficult to gain muscle even with exercise, and is considered a contributing factor to sarcopenia [7]. Understanding the age-related functional decline in myoblasts may provide insights into the complex causes of sarcopenia.

Many studies have shown that the age-related decline in skeletal muscle function, known as skeletal muscle aging, is closely linked to cellular senescence [8]. Cellular senescence is defined as irreversible cell cycle arrest that occurs when cells experience various stresses, including telomere shortening, DNA damage, and mitochondrial dysfunction [9]. Senescent cells secrete inflammatory cytokines and other factors known as the senescence-associated secretory phenotype (SASP), which promote inflammation, senescence cascades, stem cell dysfunction, and extracellular matrix alterations in neighboring cells [10]. Senescent cells accumulate with age and contribute to the pathogenesis of age-related diseases. Genetic or pharmacological clearance of senescent cells in mouse models of aging and age-related diseases has been shown to alleviate several pathological conditions [11,12]. Understanding and controlling cellular senescence provides crucial insights for developing new therapeutic strategies to prevent age-related declines in skeletal muscle function and treat age-associated diseases.

While cellular senescence is triggered by various age-related stressors, mitochondrial dysfunction is particularly implicated in skeletal muscle tissue [13,14]. Recent findings suggest that mitochondrial function is regulated by communication with other organelles. Specifically, mitochondria-ER contacts sites (MERCS) have been reported to be associated with aging phenotypes in skeletal muscle and other tissues [15,16]. Therefore, the evaluation of mitochondrial function in the context of cellular senescence should include observation of MERCS to gain a deeper understanding of the underlying mechanisms. Although studies on skeletal muscle aging, including the role of MERCS, have detailed muscle fiber phenotypes, the mitochondrial phenotype and alterations in MERCS in senescent human myoblasts remain unexplored. Because proliferating myoblasts are essential for lifelong muscle regeneration, age-associated alterations in both mitochondrial function and MERCS in this cell population may critically impair muscle repair capacity, thereby contributing to sarcopenia. We hypothesized that cellular senescence in human myoblasts is associated with distinct alterations in mitochondrial function accompanied by changes in MERCS. In this study, we aim to elucidate the mitochondrial phenotype and its relationship with MERCS in aged human myoblasts, which has not been fully characterized thus far.

## Materials and methods

### Cell culture and transfections of small interfering RNA

hSKMC (human skeletal muscle cells) were purchased from Lonza Walkersville, Inc. (CC-2561) or PromoCell GmbH (C-12530) and initiated from liquid nitrogen at the start of each experiment. The cells were cultured for 24 h to acclimate to the cell culture medium (Promocell, C-23160) and then seeded into the respective culture vessels for each assay. If necessary, the cells were transfected with small interfering RNAs (siRNAs) using Lipofectamine RNAiMAX Reagent (Invitrogen). Silencer Select siRNAs against hMFN1(s31218) and DRP1(s19559) were purchased from Thermo Fisher Scientific. Knockdown efficiency for each siRNA was confirmed by Western blotting.

### Proximity ligation assay (PLA)

PLA was performed using the Duolink In Situ Red Starter Kit Mouse/Rabbit (Duolink In Situ Detection Reagents Red (Sigma-Aldrich, DUO92008), Duolink In Situ PLA Probe Anti-Mouse MINUS (Sigma-Aldrich, DUO92004), and Duolink In Situ PLA Probe Anti-Rabbit PLUS (Sigma-Aldrich, DUO92004), according to the manufacturer’s instructions. hSKMC were fixed with 10% formaldehyde in PBS, quenched with an equal volume of 1M Glycine, and washed once with 100 mM glycine. If necessary, they were treated with MTTEMPO (Mito-TEMPO, Final 0.1 µM) for 1 h at 37 °C before fixing. After permeabilization with 0.1% Triton, hSKMC were incubated with Duolink blocking solution for 1 h at 37 °C and then incubated with primary antibodies, VDAC1 (mouse; abcam ab186321) and IP3R1 (rabbit; Invitrogen PA1–901), in Duolink antibody diluent overnight at 4 °C. Secondary antibodies PLUS and MINUS (anti-rabbit and anti-mouse IgG antibodies conjugated with oligonucleotides) were incubated 1:5 in Duolink antibody diluent for 1 h at 37 °C. The ligation solution (ligase 1:40 in ligation buffer) was then incubated for 30 min at 37 °C. The amplification solution (polymerase 1:40 in amplification buffer) was incubated for 1 h 40 min at 37 °C and then incubated with Alexa Fluor 488-phalloidin (Invitrogen, A12379) for 20 min at RT. After incubation, the slides were mounted using the Vectashield Vibrance Antifade Mounting Medium containing DAPI. PLA images were acquired using a Zeiss LSM800 (Zeiss), ZEN acquisition software, at 63 × magnification.

### Immunofluorescence

hSKMC were seeded on an 8-well chamber slide (Matsunami) and fixed with 4% Paraformaldehyde in PBS. The cells were then permeabilized for 10 min with 0.2% Triton and blocked by incubation with Blocking One Histo (Nacalai) for 1 h. After blocking, the cells were incubated for 1 h at RT with mouse monoclonal anti-TOM20 antibody (ab56783; 1:40 in PBS-T with 5% Blocking One Histo). Alexa Fluor 555 goat anti-mouse IgG (Invitrogen, A21422) secondary antibodies were incubated at a dilution of 1:500 in PBS-T with 5% Blocking One Hist for 30 min at RT and then incubated with Alexa Fluor 488-phalloidin for 20 min at RT. After incubation, the slides were mounted using the Vectashield Vibrance Antifade Mounting Medium containing DAPI. Images were acquired using Zeiss LSM800 (Zeiss), ZEN acquisition software, at 63 × magnification.

### TEM (transmission electron microscope) imaging

Young and old hSKMC were seeded on a 35 mm petri-dish (Matsunami). The cells were fixed with 2.5% glutaraldehyde (FUJIFILM Techno Products Co., Ltd., 072–02262) in a warm medium (Promocell, C-23160) for 1 h. The cells were scraped and collected using a 0.2% bovine serum albumin/0.1M PBS buffer. After washing the cells three times for five minutes in 0.1M PBS, samples were post-fixed in 1% OsO_4_ (Nisshin EM, cat no. 3028) with 1.5% potassium ferrocyanide (ScyTek Laboratories, Inc., PFD125) for 2 h. After rinsing the samples three times for five minutes with H_2_O, the cells were incubated in 1% thiocarbohydrazide (Tokyo Chemical Industry Co., Ltd, T1136) for ten minutes. After rinsing the cells with H_2_O three times, they were fixed with 1% osmium tetroxide in H_2_O for 30 min at room temperature. Samples were washed three times with H_2_O and incubated overnight with 2% uranyl acetate at 4 °C. Samples were washed three times with H_2_O at room temperature and another three times in H_2_O at 50 °C. Following the washing steps, cells were incubated in Walton’s lead at pH 5.2 (0.635% lead nitrate: FUJIFILM WakoPure Chemical Co., 124–00612, and 0.4% aspartic acid: SIGMA-ALDRICH, 01-6810-2) at 50 °C for 20 minutes. The final cell pellet was embedded in 2% low-melting-point agarose (NIPPON GENE Co., LTD., 312–06512) on ice. Agarose blocks containing the cell pellets were cut into 1–2 mm³ and dehydrated in a series of increasing ethanol concentrations (30%, 50%, 70%, and 90%) for 10 min at each concentration. A further dehydration step was performed using 100% ethanol for 10 min, three times, and twice for five minutes in a 1:1 (v/v) ethanol: acetone mixture. Finally, the samples were dehydrated thrice in pure acetone for five minutes three times at room temperature. Following the dehydration steps, resin infiltration was performed by incubating the sample in 1:1 (v/v) acetone: low-viscosity resin (Agar Scientific, AGR 1078) for 30 min with slow rotation using a tube rotator (AS ONE CO., TR-118) at room temperature. After confirming that the agar blocks sank to the bottom of the acetone/resin mixture, the samples were replaced with 100% low-viscosity resin and infiltrated for 1 h under vacuum conditions. The samples were incubated overnight in 100% resin in a vacuum chamber for further infiltration. The samples were replaced with fresh 100% resin and vacuumed for 1 h in a resin silicon mold. After orienting the sample position, resin samples were polymerized at 60 °C for 48 hours in the oven. Low Viscosity Resin mixture was prepared with the formula of LV Resin 48.0 g, VH1 Hardener 16.0 g, VH2 Hardener 36.0 g, and LV Accelerator 2.5 g. Resin blocks were trimmed with glass knives, and ultrathin 60 nm sections were made using a Diamond knife (ultra; DiATOME) in Leica Ultramicrotome (UC7). Ultrathin sections were collected on a 0.5% formvar-coated (SPI Supplies, 02463-MB) one-slot grid (EM, JAPAN) and dried. Observations and imaging were performed using a JEOL JEM-1400 FLASH 100 keV Transmission Electron Microscope.

### SA-βGal assay

Quantitation of SA-βGal activity was evaluated using Cellular Senescence Plate Assay Kit – SPiDER-βGal, a fluorogenic substrate for β-galactosidase (Dojindo), following the manufacturer’s instructions.

### Cell viability and growth assay

Cell viability and relative cell growth were assessed using the RealTime-Glo™ MT Cell Viability Assay (Promega) according to the manufacturer’s protocol. Luminescence was measured at the indicated time points, and cell growth ratio was calculated as the fold change in signal intensity relative to the initial time point (0 h), reflecting changes in viable cell number over time.

### Live cell imaging

For TMRM staining, cells were seeded in a 0.17 mm glass-bottom 10-well chamber (Greiner Bio-One, 543978). After being washed once with imaging medium (Skeletal Muscle Cell Basal Medium phenol red free; PromoCell, C-23265), they were incubated with imaging medium containing TMRM (Final 0.2 µM) and MitoBright LT Deep Red (Final 0.8 µM, Dojindo) for 30 min at 37 °C. If necessary, they were treated with FCCP (Final 10 µM) for 10 min at 37 °C after staining. The staining or FCCP medium was replaced with a fresh imaging medium. Images were acquired using Zeiss LSM800 (Zeiss), ZEN acquisition software, at 63 × magnification. For LipiRADICAL [17] (Funakoshi, FDV-0042) staining, cells were seeded in a 0.18 mm glass-bottom 8-well chamber (ibid, 80829). If necessary, they were treated with MTTEMPO (Final 10 µM) for 1 h at 37 °C before staining. After being washed once with imaging medium, they were incubated with imaging medium containing LipiRADICAL (Final 1 µM) and MitoBright LT Deep Red (Final 0.8 µM) for 1 h at 37 °C. After washing once with imaging medium, the medium was replaced with fresh imaging medium. Images were acquired using Zeiss LSM800 (Zeiss), ZEN acquisition software, at 63 × magnification.

### Mitochondrial bioenergetics

hSKMC were plated in a Seahorse XFe24 (Agilent Technologies) Extracellular Flux Analyzer culture plate in culture medium overnight. Prior to the assay, cells were switched to XF DMEM (Seahorse Biosciences) supplemented with 10 mM glucose, 2.0 mM glutamine, and 1.0 mM sodium pyruvate, following the manufacturer’s instructions. The oxygen consumption rate (OCR) during the Mito stress test was measured using a Seahorse XFe24 analyzer (Agilent) and an Extracellular Flux Analyzer (Seahorse Biosciences), following the manufacturer’s instructions. Following the Mito stress test, 1.5 µM oligomycin, 2.0 µM FCCP, and 0.5 µM antimycin A diluted in assay medium were the final concentrations in the wells.

### Gene expression analysis by real-time PCR

All reactions were performed using QuantStudio 3 (Thermo Fisher Scientific). Each sample was analyzed in triplicate for each PCR. Melting curves were checked for specificity. All mRNA expression levels were normalized to those of Actb. For quantification of mtDNA, total DNA was extracted from hSKMC using a QIAamp DNA Mini Kit (Qiagen). The following primers were used to quantify mtDNA: Actb (Hs01060665_g1), Nd1 (Hs02596873_s1), Nd5 (Hs02596878_g1), and Cytb (Hs02596867_s1), all of which were purchased from Thermo Fisher Scientific.

### Western blotting

hSKMC were lysed by RIPA A buffer (Wako) supplemented with Halt protease and phosphatase inhibitor cocktail and EDTA solution for 30 min at 37 °C, and the supernatant was collected after centrifugation (15,000 rpm for 5 min at 4°C). The cell lysates were quantified using the Pierce BCA Protein Assay kit (Thermo Fisher Scientific), separated by 5–20% SDS-PAGE, and blotted onto Immobilon PVDF membranes (IPVH07850, Millipore). Blots were probed with the indicated antibodies, and protein bands were visualized using Amersham^TM^ ECL^TM^ Prime (Cytiva). Band images were captured using FUSION SOLOS (Vilber-Lourmat). Relative band intensities were quantified using the Fiji/ImageJ software.

### Proteomic analysis

Samples were digested with phase-transfer surfactants according to previously reported methods [18] and subjected to liquid chromatography-tandem mass spectrometry (LC–MS/MS) analysis using an UltiMate 3000 Nano LC system (Thermo Fisher Scientific) coupled with an Orbitrap Exploris 480 mass spectrometer (Thermo Fisher Scientific) for data-independent acquisition. Samples were injected by an autosampler and enriched on a C18 reverse-phase trap column (100 μm inner diameter × 5 mm length; Thermo Fisher Scientific) at a flow rate of 8 μL/min. They were subsequently separated on an IonOpticks 25 cm Aurora column (Bruker Daltonics, Fitzroy, VIC, Australia) at a flow rate of 300 nL/min with a linear gradient from 3% to 35% mobile phase B. Mobile phase B consisted of acetonitrile with 0.1% formic acid, and mobile phase A consisted of distilled water with 0.1% formic acid. The peptides were ionized using nano-electrospray ionization in the positive ion mode. A spray voltage of 2,100 V was applied. MS1 spectra were collected in the m/z range of 497–740 at 15,000 resolution to set an auto-gain control (AGC) target of 3 × 10^6. MS2 spectra were collected in the m/z range of 200–1,800 at 45,000 resolution to set an AGC target of 3 × 10^6, automatic maximum injection time, and stepped normalized collision energies of 22%, 26%, and 30%. Data-independent acquisition (DIA) data were analyzed using DIA-NN (version 1.8.1) against the UniProt human database (https://www.uniprot.org; downloaded February 2022 with 20,577 sequences) in the robust liquid chromatography (high precision) mode with retention time–dependent cross-run normalization. The enzyme trypsin was set to be fully specific, allowing for one missed cleavage. Both Mass and MS1 accuracies were set to 10 ppm, a match between runs was enabled, and the precursor false discovery rate was controlled at 1%. Carbamidomethylation of cysteine was set as a fixed modification, and oxidation of methionine was set as a variable modification.

### Antibodies and chemicals

#### Antibodies.

Rabbit polyclonal anti-Lamin B1 antibody (ab16048, 1:2000) was purchased from Abcam. Rabbit polyclonal b-Tubulin antibody (2146, 1:5000), rabbit monoclonal p16 antibody (80772, 1:2000), rabbit monoclonal DRP1 antibody (8570, 1:2000), and rabbit monoclonal MFN1 antibody (14739, 1:2000) were purchased from Cell Signaling.

#### Chemicals.

FCCP (0453) was purchased from Tocris Bioscience. MTTEMPO (SML0737) and TMRM (T5428) were purchased from Sigma-Aldrich.

### Image processing, analysis, and statistics

Mitochondrial morphology was analyzed using the plugin, and MiNA morphology was analyzed using StuartLab from the ImageJ/Fiji Software. The PLA images were analyzed manually with ‘Threshold’ and ‘Analyze particles’ commands from ImageJ/Fiji Software, and PLA dot areas were normalized on each cell area. The percentage of MERCS from Electron Microscopy images was measured as the ratio of the mitochondria-ER distance of <25 nm lengths the mitochondrial perimeter. The lengths and perimeters were analyzed manually using ImageJ/Fiji Software. The intensity of LipiRADICAL staining was measured as the integrated density of each cell type. We measured the intensity of TMRM in only the mitochondrial area, analyzed manually with ‘Threshold’ and ‘Analyze particles’ commands from ImageJ/Fiji Software from the MitoBright LT Deep Red images. Statistical significance between two groups was determined using a standard unpaired two-sided t-test with Welch’s t-test. The statistical significance between multiple groups was determined by one-way analysis of variance (ANOVA) to ensure comparable variance, and comparisons for controls were performed using Dunnett’s test. The analysis was done in Prism 10, GraphPad. The asterisk represents a *p*-values, *p < 0.05, **p < 0.01, ***p < 0.001, and **** *p* < 0.0001. No statistical methods were used to determine the sample sizes. The investigators were not blinded to the allocation during the experiments or outcome assessment. Because imaging quantification was not performed under blinded conditions, the possibility of measurement bias cannot be excluded.

### Ethics statement

Primary human skeletal muscle cells used in this study were obtained commercially from PromoCell GmbH and Lonza Group Ltd., which collected donor tissue under informed written consent and with approval from the appropriate ethics committee. All samples were fully anonymized by the supplier prior to distribution. Because the authors used only commercially available, fully anonymized human primary cells, additional ethics approval and participant consent for this research were not required, in accordance with the institutional guidelines and PLOS ONE human subjects research policies.

## Result

### Human skeletal muscle myoblasts from elderly donors exhibit a senescence phenotype

**Fig 1:**
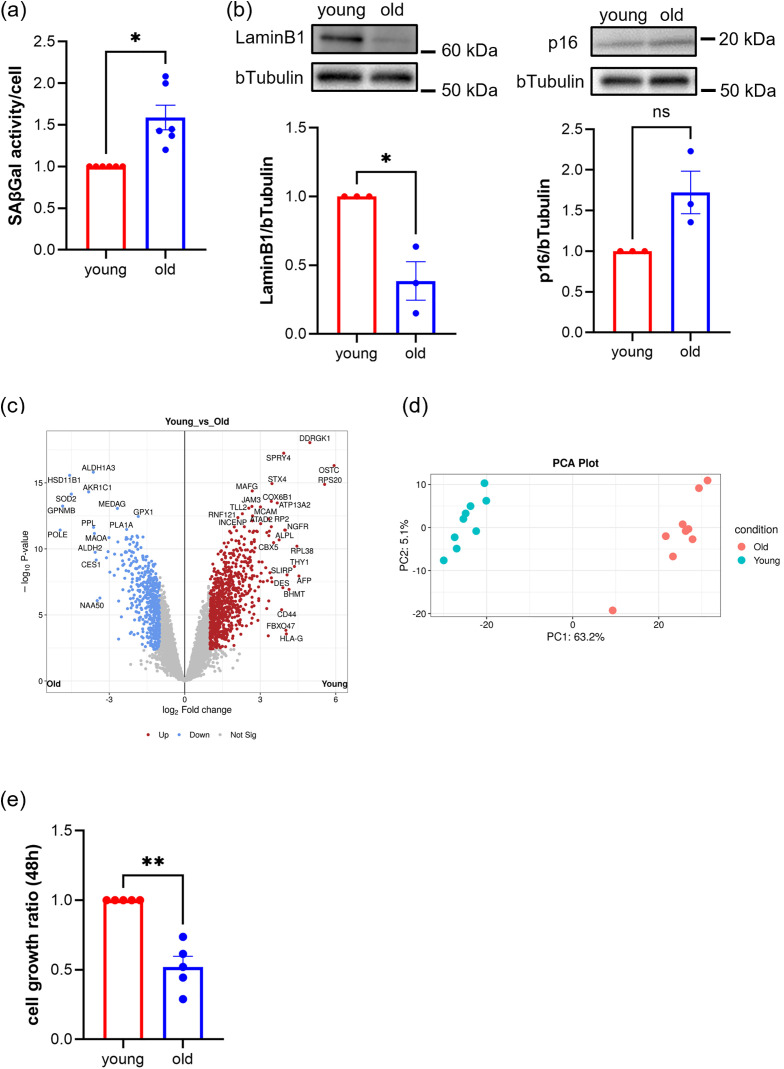
Human skeletal muscle myoblasts derived from elderly donors exhibit a senescent phenotype. **(a)** Results of SA-βGal activity measurement per cell (n = 3 biological replicates; cells counted: young, 6, old, 6). **(b)** Western blot analysis showing detection of Lamin B1 and p16 (n = 3 biological replicates; cells counted: young, 3, old, 3). **(c)** Blue dots in the volcano plot indicate proteins whose expression increased in aged cells; red dots indicate proteins whose expression increased in young cells (n = 9 biological replicates). **(d)** The PCA plot shows the similarity between samples from each group. **(e)** Changes in relative cell growth over 48 hours of culture, assessed by a viability-based assay (n = 3 biological replicates; cells counted: young, 5, old, 5). All values are expressed relative to the activity of young cells, which is set to 1. The *p*-values were calculated using Welch’s t-test. *; *p* < 0.05, **; *p* < 0.01.

We compared commercially available human skeletal muscle myoblasts derived from young and elderly donors to determine whether these cells from elderly donors exhibit a senescence phenotype. For all experiments, myoblasts from healthy Caucasian male donors aged 20–30 years and 60–90 years were used as young and old cells, respectively. This study used commercially available human skeletal muscle cells, and therefore ethics approval was not required. To prevent changes in myoblast characteristics due to long-term culture or repeated passages, fresh cell stocks stored in liquid nitrogen were used for each experiment. Passaging was limited to one passage, and the total culture period was maintained at 5 days [19,20]. Examination of SA-βGal activity, Lamin B1, and p16 expression in young and old cells revealed that SA-βGal activity was significantly increased, while Lamin B1 was significantly decreased in old cells (Fig 1a and 1b). In addition, p16 protein levels showed an increasing trend in old myoblasts, although this difference did not reach statistical significance (Fig 1b). Proteomic analysis of whole cell lysates from young and old cells showed enhanced oxidative stress responses in old cells, as indicated by increased expression of SOD2 and ALDH1A3 (retinaldehyde dehydrogenase 3). In contrast, young cells have higher levels of proteins involved in protein synthesis, cell proliferation, and signal transduction, such as DDRGK1(DDRGK domain-containing protein 1), SPRY4 (protein sprouty homolog 4), and RPS20 (small ribosomal subunit protein uS10), indicating their higher proliferative capacity (Fig 1c). Additionally, the PCA plot results showed that young and old cell groups could be distinctly categorized, validating their suitability for aging phenotype analysis (Fig 1d). Old cells exhibited reduced cell growth compared to young cells (Fig 1e), further confirming the senescent phenotype. These findings indicate that myoblasts from the elderly donors used in this study displayed a typical senescence phenotype of proliferative stem cells.

### Human skeletal muscle myoblasts from elderly donors do not show decline in mitochondrial function

**Fig 2:**
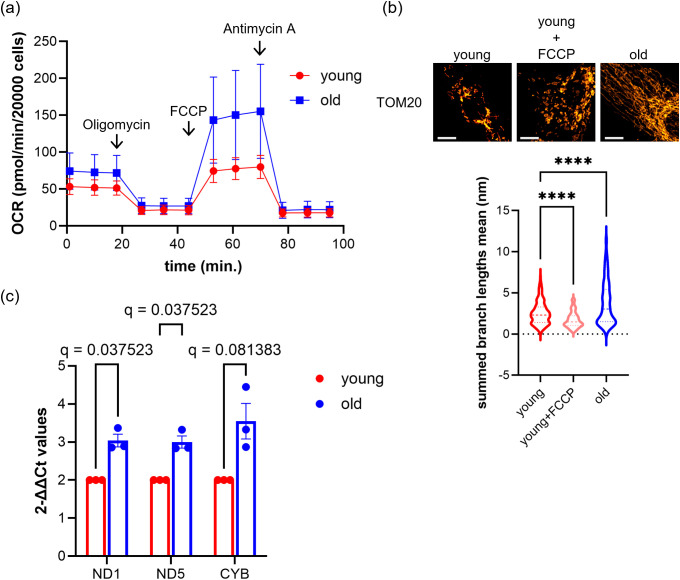
Human skeletal muscle myoblasts derived from elderly donors do not show a decline in mitochondrial function. **(a)** Results of oxygen consumption measurements using the Seahorse Flux Analyzer. Each point represents the mean ± **S.**D. deviation for young and old cells (n = 3 biological replicates), with 20,000 cells per measurement. **(b)** Results of mitochondrial morphology. The images are partial views of cells stained with TOM20 antibody, with the scale bar representing 10 μm. The graph shows the average mitochondrial size per cell measured from the images, presented as violin plots (n = 3 biological replicates; cells counted: young, 239, young+FCCP, 220, old, 264). *p*-values were calculated using Dunnett’s test with young as the control, **; *p* < 0.01, ***; *p* < 0.001. **(c)** Results of mitochondrial DNA quantification (n = 3 biological replicates; cells counted: young, 3, old, 3). The quantity of mitochondrial genes was normalized using bAct as an internal standard. q-values were calculated using Welch’s t-test for multiple comparisons.

Next, we evaluated the mitochondrial energy metabolism in old cells using a Seahorse Flux Analyzer to measure oxygen consumption. The results demonstrated an increased oxygen consumption in old cells (Fig 2a). Additionally, immunostaining revealed excessive mitochondrial fusion in old cells (Fig 2b), suggesting an increased mitochondrial mass. Importantly, the increased oxygen consumption in old cells was accompanied by a preserved increase in maximal respiration following FCCP treatment (Fig 2a), arguing against a state of pre-existing mitochondrial uncoupling. If mitochondria were already substantially uncoupled, FCCP would not be expected to further elevate oxygen consumption. In addition, FCCP treatment induced marked mitochondrial fragmentation in young cells (Fig 2b), whereas mitochondria in old cells did not display a comparable fragmented morphology, further supporting that elevated OCR in old cells is unlikely to reflect a pre-uncoupled state. Therefore, to determine whether mitochondrial quantity explains the elevated oxygen consumption, we quantified mitochondrial DNA. Mitochondrial DNA content was assessed by quantifying ND1 (NADH-ubiquinone oxidoreductase chain 1), ND5 (NADH-ubiquinone oxidoreductase chain 5), and CYB (cytochrome b). As anticipated, mitochondrial content increased in old cells (Fig 2c), suggesting a correlation with increased oxygen consumption. However, according to proteomics data, there was no change in the expression of respiratory enzymes (S1a in S1 Fig). We further quantified membrane potential per mitochondrial area. Staining with MT Bright Deep Red and TMRM allowed for the evaluation of membrane potential intensity relative to mitochondrial quantity, with no significant difference observed between young and old cells (S1b in S1 Fig). Thus, we did not observe a decline in key aspects of mitochondrial function in myoblasts from elderly donors.

### Increased MERCS in human skeletal muscle myoblasts from elderly donors

**Fig 3:**
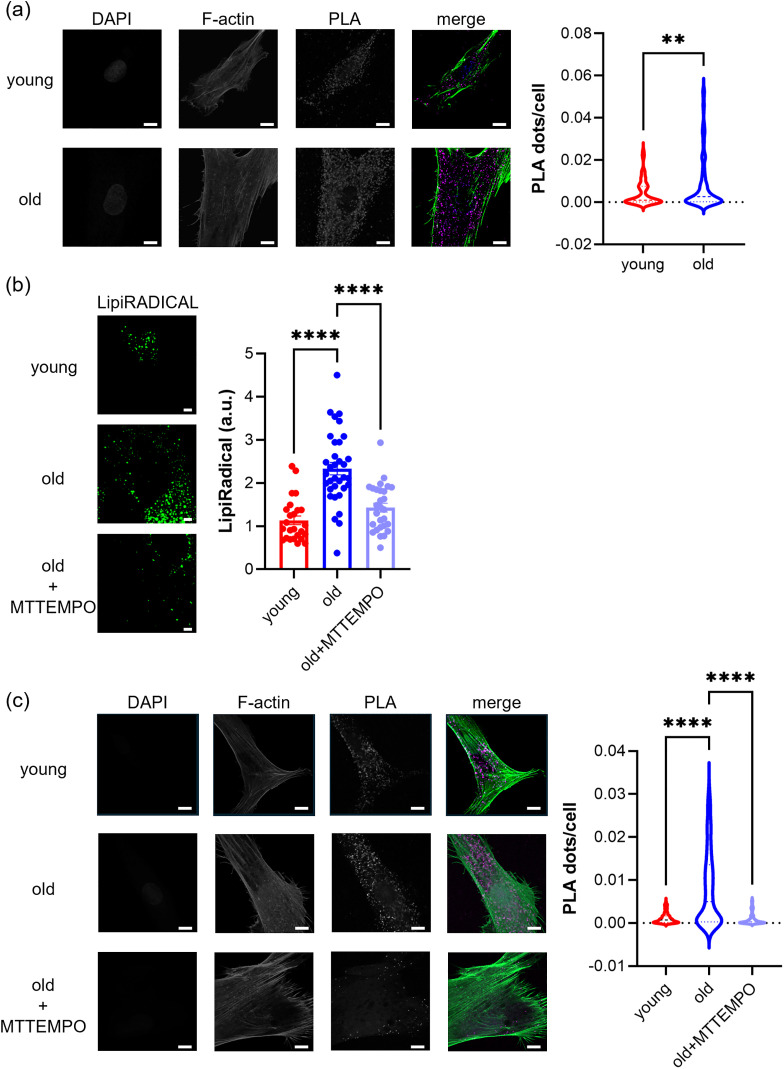
MERCS are increased in human skeletal muscle myoblasts derived from elderly donors. **(a)** Detection of MERCS using proximity ligation assay (PLA) with IP3R1 and VDAC1 antibodies. The area of PLA dots was normalized to the area of the cell to obtain quantitative values, which are presented as violin plots (n = 3 biological replicates; cells counted: young, 115, old, 139). The scale bar represents for 10 μm. *p*-values were calculated using Welch’s t-test, ****; *p* < 0.0001. **(b)** Detection of lipid peroxides using LipiRADICAL Green. The scale bar represents 5 μm. The graph quantifies the intensity values, plotting the average per cell (n = 3 biological replicates; cells counted: young, 25, old, 34, old+MTTEMPO, 28). Error bars represent standard deviation. *p*-values were calculated using Dunnett’s test with young as the control, ****; *p* < 0.0001. **(c)** Detection of MERCs using PLA as in **(a)** (n = 3 biological replicates; cells counted: young, 228, old, 167, old+MTTEMPO, 133). *p*-values were calculated using Dunnett’s test with old as the control, ****; *p* < 0.0001.

MERCS are associated with various mitochondrial functions, such as bioenergetic regulation via calcium influx [21] and the removal of lipid peroxides through reactive oxygen species [22]. Given that mitochondrial function was maintained in old cells, we examined whether age-associated changes in MERCS were present and therefore quantified MERCS. The results indicated a significant increase in MERCS in old cells (Fig 3a and S2a in S2 Fig). However, proteomic data revealed no changes in the expression levels of tethering proteins in MERCS (S2b in S2 Fig). Considering the proteomics results showing increased SOD2 expression in old cells (Fig 1c) and reports that MERCS increase in oxidatively stressed mitochondria [22], we hypothesized that mitochondrial oxidative stress contributes to increased MERCS in old cells. Lipid peroxide detection using LipiRADICAL Green showed a significant increase in peroxide levels in old cells (Fig 3b). Moreover, suppression of mtROS by MTTEMPO reduced peroxide generation (Fig 3b). Furthermore, reducing mitochondrial oxidative stress in old cells treated with MTTEMPO resulted in a decrease in MERCS (Fig 3c). These findings suggest that mitochondria in old cells are in an oxidative state and that increased MERCS may be involved in cellular responses to mitochondrial oxidative stress.

## Discussion

We investigated the mitochondrial function and MERCS in primary cultured myoblasts derived from the human skeletal muscle of young and elderly donors in relation to cellular senescence. Myoblasts from elderly donors exhibited a senescence phenotype, including increased SA-βGal activity and decreased Lamin B1 expression [23], consistent with established markers of cellular aging. In addition, p16 protein levels tended to be higher in aged myoblasts, although the difference did not reach statistical significance. The assessment of p21 was not included due to its pronounced inter-donor variability in primary human myoblasts, limiting its reliability as a senescence marker in this context. In this study, aged myoblasts also displayed mitochondrial hyperfusion and increased MERCS.

Mitochondrial hyperfusion has also been reported in senescent or low-proliferative states [24], although its functional significance remains incompletely understood. Since mitochondrial energy metabolism is not impaired in old myoblasts, no decline was observed in key aspects of mitochondrial bioenergetic function. This observation is consistent with the idea that fused mitochondrial networks may support bioenergetic stability under stress [25]. Fragmented mitochondria are typically associated with higher proliferative capacity [26]. Because mitochondrial dynamics influence ROS levels in multiple cell types [27], the hyperfusion observed in aged myoblasts may reflect reduced proliferation and altered redox status rather than a direct functional adaptation.

Proliferating stem-like cells generally favor mitochondrial fission [28]. Consistent with this, our data showed that the proliferation capacity of old cells was approximately half that of young cells (Fig 1e), and that a fused mitochondrial phenotype was associated with impaired proliferation. Furthermore, altering mitochondrial dynamics through MFN1 or DRP1 knockdown did not restore proliferative capacity (S3 Fig). These results support the view that mitochondrial morphology reflects proliferative status rather than directly determining it, as induction of fission in old cells did not restore proliferation to levels comparable to those in young cells. This finding calls into question a simplistic model in which mitochondrial fission directly promotes proliferation and fusion merely reflects senescence. Instead, our data supports a more nuanced view in which mitochondrial morphology represents a downstream marker of the cellular state, shaped by proliferative capacity and metabolic context rather than acting as a primary determinant.

In contrast to typical age-related mitochondrial decline observed in skeletal muscle tissue [14,29], these features were not evident in our primary myoblast cultures. These differences may reflect the distinct metabolic states of proliferating myoblasts, which rely heavily on aerobic glycolysis [30]. Under such glycolytic conditions, mitochondrial impairments may be functionally masked, and fusion-dominant networks may further buffer metabolic deficits [29]. This metabolic context may explain why mitochondrial deficits were not phenotypically detectable in aged myoblasts.

Although age-associated reductions in MERCS have been reported in skeletal muscle tissue [31,32], aged myoblasts in our study showed increased MERCS. Given that MERCS can be induced by oxidative stress [20], the increase observed here may reflect elevated mtROS rather than chronological age. Consistently, scavenging mtROS with MTTEMPO reduced MERCS abundance. Because oxidative stress responses vary across cell types and culture conditions, direct comparison with in vivo muscle tissue remains challenging. Moreover, we found that altering mitochondrial fission or fusion via siRNA did not change MERCS abundance (S4 Fig). This result suggests that MERCS are not solely determined by mitochondrial morphology. One possible scenario is that increased mitochondrial oxidative stress in aged human myoblasts is associated with enhanced MERCS formation. Elevated mtROS may influence mitochondria–ER proximity, potentially impacting mitochondrial homeostasis through mechanisms such as calcium signaling or lipid metabolism. At the same time, oxidative stress may impair the regulation of MERCS tethering, leading to altered or dysregulated contact formation. Further studies will be required to determine the causal mechanisms underlying mtROS-dependent MERCS remodeling.

Although mitochondrial function and fiber-type composition differ substantially among skeletal muscles in vivo, the present study analyzed proliferating primary human myoblasts rather than terminally differentiated myofibers. As these cells had not undergone myogenic differentiation, they do not exhibit fast- or slow-twitch phenotypes or fiber-type–specific mitochondrial specialization. Therefore, the observed phenotypes are more likely to reflect cell-intrinsic aging-associated responses under uniform in vitro conditions rather than muscle-type–specific metabolic programs. Nevertheless, we acknowledge that the use of myoblasts derived from different skeletal muscle sources represents a limitation of the study. Most studies of muscle aging have focused on mature myofibers, and mitochondrial physiology in proliferating myoblasts remains less defined. Therefore, in vitro myoblast phenotypes may differ substantially from in vivo muscle tissue. Mitochondrial function and MERCS regulation likely depend on cellular metabolic state, proliferative capacity, and tissue context. Our findings indicate that aged myoblasts exhibit mitochondrial hyperfusion and increased MERCS under elevated oxidative stress, potentially supporting mitochondrial stability in a stress-adapted state. Further studies across differentiation stages and cell types will help clarify how mitochondrial dynamics and MERCS contribute to muscle aging.

## Supporting information

S1 Fig Expression of respiratory enzymes and membrane potential measurement in skeletal muscle myoblasts. (DOCX)

S2 Fig TEM imaging of MERCs and expression levels of tethering proteins in skeletal muscle myoblasts. (DOCX)

S3 Fig Changes in Mitochondrial Morphology and Cell Proliferation Rate in Skeletal Muscle Myoblasts using siRNA. (DOCX)

S4 Fig MERCs do not change with mitochondrial morphology changes. (DOCX)

S5 Fig Original uncropped and unadjusted blot images corresponding to Fig 1b and S3a Fig. (DOCX)
