# Supplementary material for: Characterizing mitochondrial phenotypes and MERCS in aged human skeletal muscle myoblasts
Source: PLoS One. 2026 Feb 20;21(2):e0343604. doi: 10.1371/journal.pone.0343604 (PMC12923047; doi:10.1371/journal.pone.0343604)
Supplement: S1 Fig — (DOCX) [file pone.0343604.s001.docx]

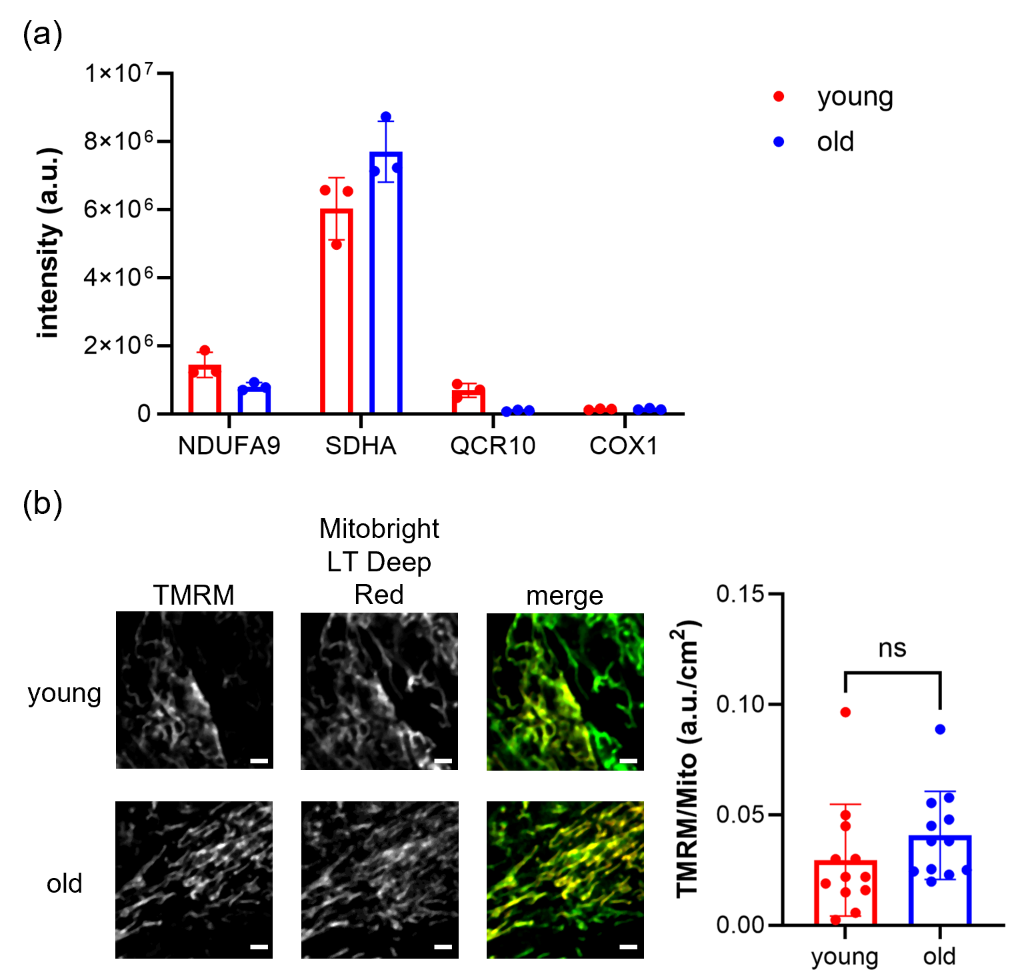


S1 Fig. Expression of respiratory enzymes and membrane potential measurement in skeletal muscle myoblasts.

(a) Proteomics analysis data showing the expression levels of mitochondrial respiratory enzymes. NDUFA9 represents subunits of Complex I, SDHA of Complex II, QCR10 of Complex III, and COX1 of Complex IV. Each plot represents the mean ±S.E (n = 3 biological replicates). (b) Results of membrane potential measurement per mitochondrial area. Cells were stained with MitoBright LT Deep Red and TMRM and detected using confocal microscopy. The scale bar represents 2.5 μm. Each plot represents the mean ± S.D (n = 3 biological replicates; cells counted: 12). p-values were calculated using Welch’s t-test.
