# Supplementary material for: Characterizing mitochondrial phenotypes and MERCS in aged human skeletal muscle myoblasts
Source: PLoS One. 2026 Feb 20;21(2):e0343604. doi: 10.1371/journal.pone.0343604 (PMC12923047; doi:10.1371/journal.pone.0343604)
Supplement: S2 Fig — (DOCX) [file pone.0343604.s002.docx]

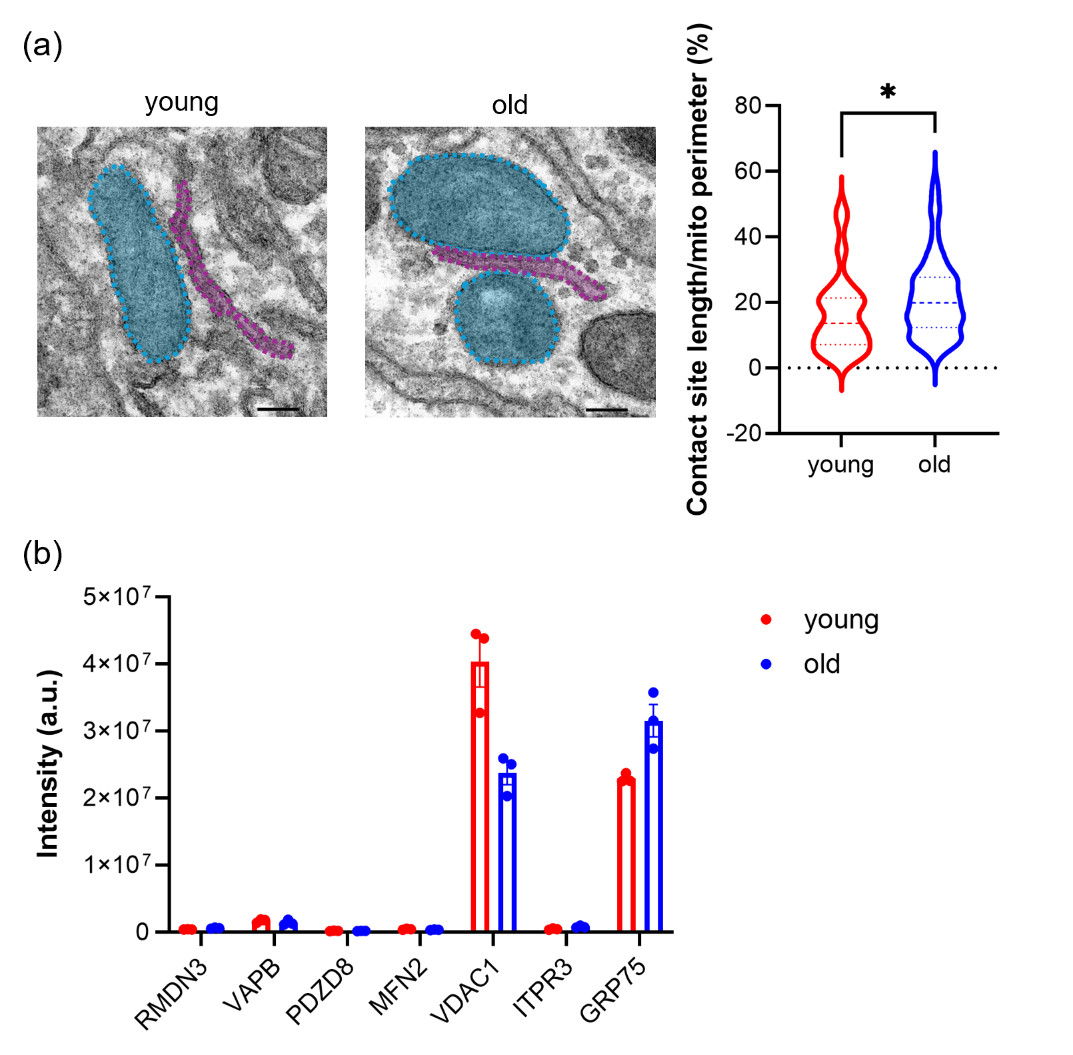


S2 Fig. TEM imaging of MERCs and expression levels of tethering proteins in skeletal muscle myoblasts.

(a) The images are sections of cells captured using TEM. Distances less than 25 nm between blue mitochondria and purple ER were measured and defined as contact site lengths. The graph shows quantitative values per mitochondria from five different cells in both young and old cells, presented as violin plots (n = 3 biological replicates; cells counted: young, 41, old, 124). *p*-values were calculated using an unpaired t-test. * *p*<0.05. (b) Expression levels of well-known tethering proteins that constitute MERCs were analyzed using proteomics (n = 3 biological replicates). No significant differences were found in the multiple comparison tests using Welch's t-test.
