# Supplementary material for: Characterizing mitochondrial phenotypes and MERCS in aged human skeletal muscle myoblasts
Source: PLoS One. 2026 Feb 20;21(2):e0343604. doi: 10.1371/journal.pone.0343604 (PMC12923047; doi:10.1371/journal.pone.0343604)
Supplement: S3 Fig — (DOCX) [file pone.0343604.s003.docx]

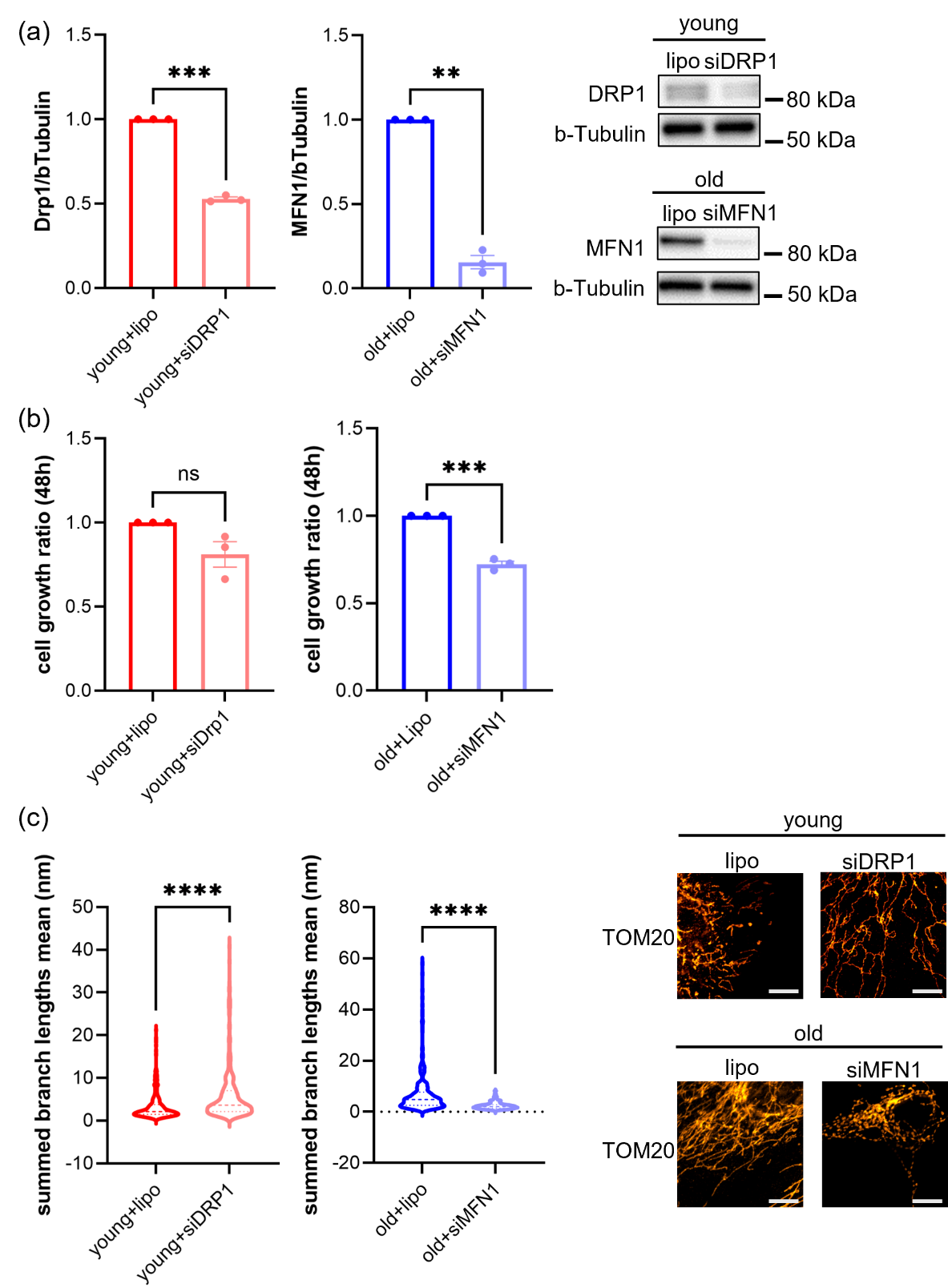


S3 Fig. Changes in Mitochondrial Morphology and Cell Proliferation Rate in Skeletal Muscle Myoblasts using siRNA.

(a) Western blot analysis showing protein expression levels of DRP1 in young cells and MFN1 in old cells after their expression was reduced by siRNA. The results are presented as relative values, with the control cells treated with lipofectamine (+lipo) set to 1 (n = 3 biological replicates). (b) Changes in relative cell growth after reducing the expression of DRP1 in young cells and MFN1 in old cells using siRNA, assessed by a viability-based assay (n = 3 biological replicates). (c) Quantification of mitochondrial morphology after reducing the expression of DRP1 in young cells and MFN1 in old cells using siRNA (n = 3 biological replicates; cells counted: young+lipo, 286, young+siDRP1, 319, old+lipo, 308, old+siMFN1, 284). *p*-values were calculated using Welch's t-test, ** *p*<0.01, *** *p*<0.001, **** *p*<0.0001.
