# Supplementary material for: Characterizing mitochondrial phenotypes and MERCS in aged human skeletal muscle myoblasts
Source: PLoS One. 2026 Feb 20;21(2):e0343604. doi: 10.1371/journal.pone.0343604 (PMC12923047; doi:10.1371/journal.pone.0343604)
Supplement: S4 Fig — (DOCX) [file pone.0343604.s004.docx]

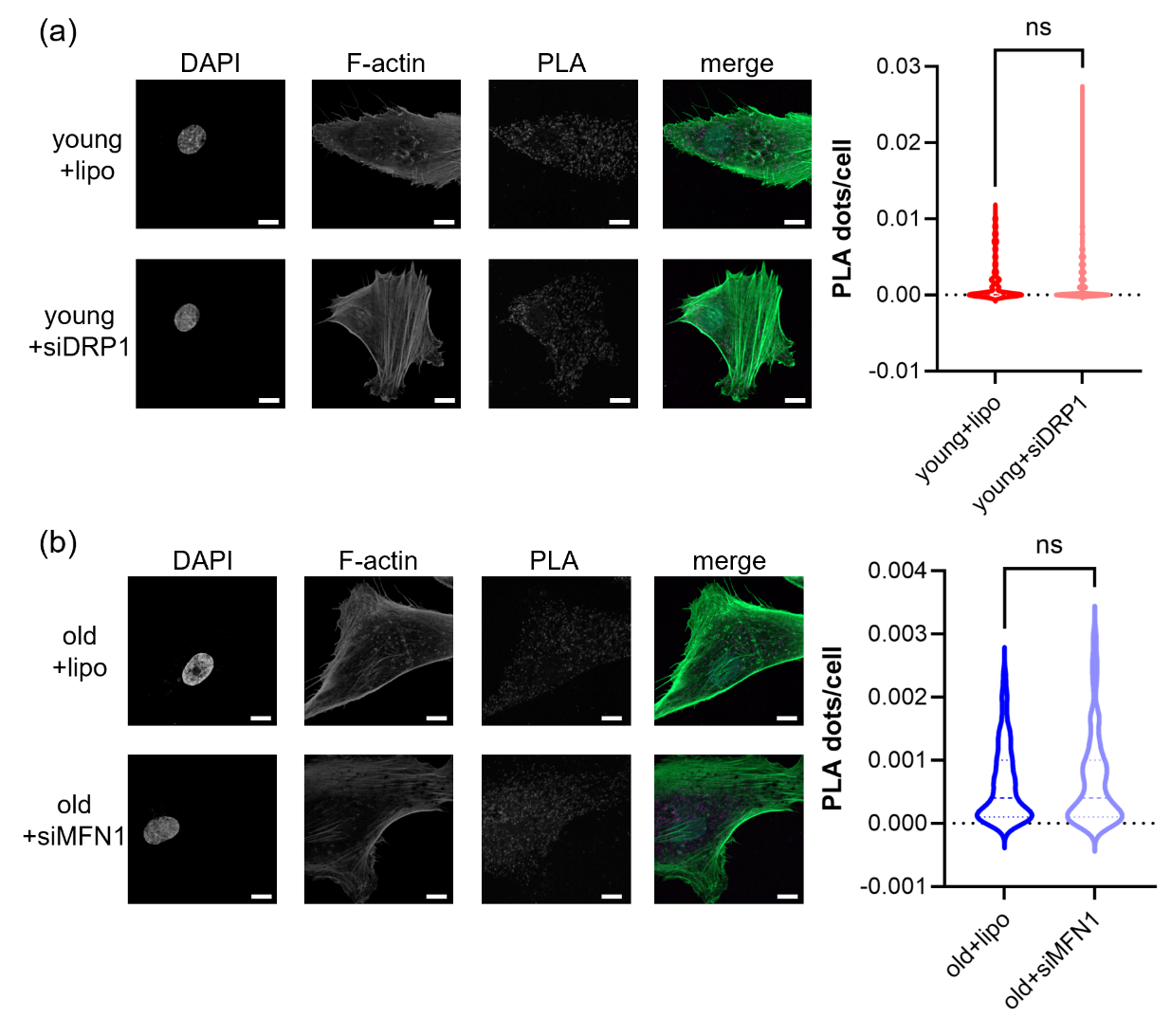


S4 Fig. MERCs do not change with mitochondrial morphology changes.

Detection of MERCs using proximity ligation assay (PLA) with IP3R1 and VDAC1 antibodies. The area of the PLA dots was normalized to the area of the cell to obtain quantitative values, which are presented as violin plots: (a) n = 3 biological replicates; cells counted: young+lipo, 334, young+siDRP1, 398, and (b) n = 3 biological replicates; cells counted: old+lipo, 176, old+siMFN1, 174. Scale bar represents 10 μm. *p*-values were calculated using Welch’s t-test.
