# Supplementary figures and images for: Characterizing mitochondrial phenotypes and MERCS in aged human skeletal muscle myoblasts
Source: PLoS One. 2026 Feb 20;21(2):e0343604. doi: 10.1371/journal.pone.0343604 (PMC12923047; doi:10.1371/journal.pone.0343604)

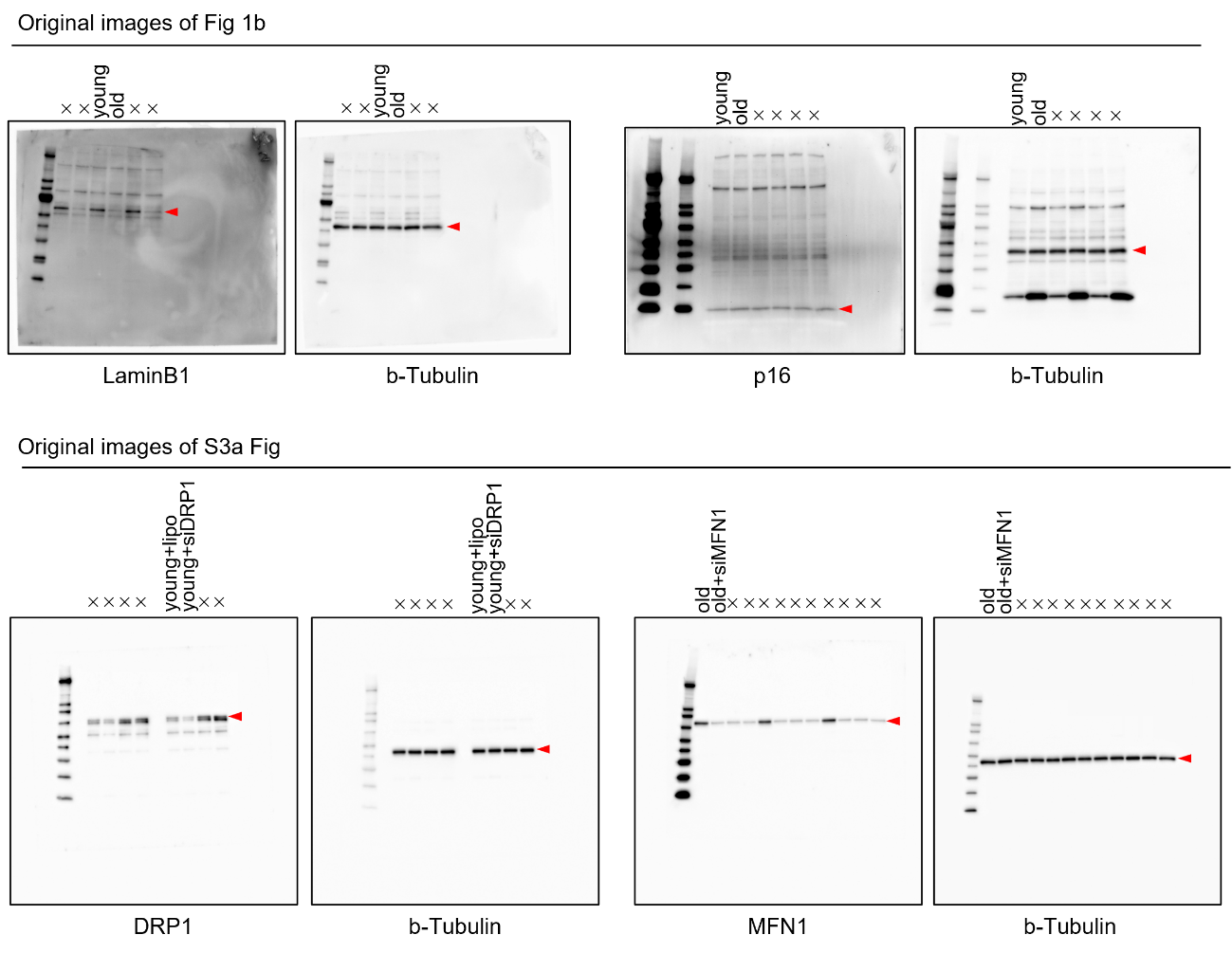


S5 Fig. Original uncropped and unadjusted blot images corresponding to Fig 1b and S3a Fig.

Supplement: S5 Fig — (DOCX) [file pone.0343604.s005.docx]
